# Monthly Summary

**Published:** 1888-07

**Authors:** 


### Monthly Summary.

---

Blind Abscess.—Dr. W. H. Atkinson, in N. Y. Society.—A blind abscess should be treated like blind people. Give them eyes; give them vent. Open them up to the disinfecting influences of the air and what else you please. Hew down into it and clear out the passages, and don't you forget it. Let all your mugwumpian ideas of filling go by the board. Be sure you are right and go straight to effect your purpose. What is disinfecting? What are antiseptics? We have heard a great deal about them. All the substances named are simply agents that holds oxygen with a loose grip, so they can carry it into the territory where the greater vacancy exists and leave it on the ground. That is the sesame of the whole thing—

this idea of holding the oxygen loosely and carrying it to the place in which it will do most good.

One thing that seems to haunt the minds of most of the speakers has not been clearly set forth—how to fill at root that is curved, the foramen of which is larger than the main canal. Our friend Dwinelle puts a hook up there, finds were the end of the root is, and takes a measure. I say measure so that you shall not go through the end, and then with a stiff wire bur, made a little shorter than the tooth, carry the material to place.

One thing more, and that is, do not be afraid of mischief coming about from microbes and those kind of things. Green apples give the boys the bellyache, and those disturbing elements, the microbes, they say, gave us the toothache. How much do we know about microbes? Do we know anything of their life history? Do we know anything of the results that will follow if we let them alone? Are they always poison, or do they sometimes do good? They say there must be microbes to make pus possible. Pus is dead blood. When the scrum is killed it is not capable of being built into tissue. Suppose you have filled a tooth, and there comes a blind abscess, what have you got to do? Just cut right into the seat of the mischief, and let me say, gentlemen, if I never see you again, you had better cut fifty times when you do not need to, and then to forget to cut once when you do. When a pulp is dead you can cut through gum, alveolar wall and all, and be sure you are not doing mischief.—*Odontographic Journal.*

---

Destroying Pulps.—Dr. C. J. Tibbets recommends *arsenite of potassa* for destroying the vitality of tooth-pulp. The preparation is made as follows: take twelve grains of caustic potash, ten grains of arsenious acid, place in a mortar and add a few drops of water to assist in reducing the potash and arsenic to a thick, creamy paste, then add ten grains of sulphate of morphia (or muriate cocaine) and stir for fifteen or twenty minutes to prevent re-crystallization; keep in wide-mouthed bottles, well stopped with cork boiled in paraffine." In applying the paste the same precautions are to be used as with arsenic. The cavity is sealed with gutta-percha, incorporated with enough wax to render it non-plastic. The arsenite of potassa may be applied to the pulp of an aching tooth, it matters not how great the congestion may be, and in from three to twelve hours the pulp may be entirely removed. If the dressing be undisturbed for ten or twelve days, most frequently no remains of the pulp will be found save a soapy condition of the chamber and root canals, rendering the subsequent cleansing a very easy matter. No greater pain will follow the application than that which caused the visit.—*Chirurgical Dental Journal.*

---

SWAGING MACHINE.—Mr. L Matheson after experimenting with the Tauber Hydraulic Press says: The object of the invention is to supercede the ordinary zinc and lead casts and dies in the making of metal dentures. by the employment of hydraulic pressure, applied directly to the plate, resting upon a cast of Spense metal—a curious compound which it is possible to pour, whilst molten, into a Stent or gutta-percha impression, but which, when cool, is quite hard enough to bear a strain greater than zinc. Two claims are made for the machine. (1) That a great saving of time is effected by doing away with the necessity for the ordinary casts and dies; and (2) that a good fit is obtained with more ease and certainty than by the method now in use, owing the principle of the appliance, by which pressure is brought to bear equeally on all parts.—*British Journal of Dental Science.*

